# Cardiac damage after treatment of childhood cancer: A long-term follow-up

**DOI:** 10.1186/1471-2407-8-141

**Published:** 2008-05-20

**Authors:** Veronika Velensek, Uros Mazic, Ciril Krzisnik, Damjan Demšar, Janez Jazbec, Berta Jereb

**Affiliations:** 1 University Children's Hospital Ljubljana, Vrazov trg 1, Ljubljana, Slovenia; 2 Institute of Oncology, Zaloška 2, Ljubljana, Slovenia; 3 Institut "Jožef Stefan", Jamova cesta 39, 1000 Ljubljana, Slovenia

## Abstract

**Background:**

With improved childhood cancer cure rate, long term sequelae are becoming an important factor of quality of life. Signs of cardiovascular disease are frequently found in long term survivors of cancer. Cardiac damage may be related to irradiation and chemotherapy.

We have evaluated simultaneous influence of a series of independent variables on the late cardiac damage in childhood cancer survivors in Slovenia and identified groups at the highest risk.

**Methods:**

211 long-term survivors of different childhood cancers, at least five years after treatment were included in the study. The evaluation included history, physical examination, electrocardiograpy, exercise testing and echocardiograpy. For analysis of risk factors, beside univariate analysis, multivariate classification tree analysis statistical method was used.

**Results and Conclusion:**

Patients treated latest, from 1989–98 are at highest risk for any injury to the heart (73%). Among those treated earlier are at the highest risk those with Hodgkin's disease treated with irradiation above 30 Gy and those treated for sarcoma. Among specific forms of injury, patients treated with radiation to the heart area are at highest risk of injury to the valves. Patients treated with large doses of anthracyclines or concomitantly with anthracyclines and alkylating agents are at highest risk of systolic function defect and enlarged heart chambers. Those treated with anthracyclines are at highest risk of diastolic function defect. The time period of the patient's treatment is emerged as an important risk factor for injury of the heart.

## Background

While agressive anticancer therapy in children is increasingly successful in terms of survival, its adverse effects are becoming more apparent. In childhood cancer the survivor life expectancy is long and the impact of late sequelae on their life quality is high [1]. These patients are one of the largest risk groups for cardiovascular diseases, the cardiac injury being related both to chemotherapy, especially with anthracyclines, and irradiation [2,3]. The incidence and severity of cardiomyopathy depend on the cumulative dose of anthracyclines. The tolerance for anthracyclines is individual and cardiomyopathy may progress for years after discontinuation of therapy. Cardiotoxicity may be worsened by additional risk factors such as the patient age, female sex, type of cancer, radiation therapy to fields that involve heart and concomitant exposure to cyclophosphamide [2,4-10].

The purpose of the present study was: 1) to evaluate simultaneous influence of a series of independent variables on late cardiac damage in childhood cancer survivors in Slovenia using noninvasive evaluation methods; 2) to identify groups of patients that are at the highest risk for late cardiac damage.

## Patients and Methods

### Patients

In Slovenia, a single center serves as a national referral center for all children with malignant disorders. After the end of treatment, all children are followed at the center for at least five years or until they are 18 years old. Later, they are followed regularly at the outpatient Clinic for Late Effects at the Institute of Oncology, Ljubljana. [11].

According to the Cancer Registry of Slovenia, between 1968 and 1998 1744 children were treated for malignant diseases at the Oncology and Hematology Department of the University Children's Hospital, Ljubljana and at the Institute of Oncology, Ljubljana. Of the 874 survivors, 399 were regularly followed at the outpatients Clinic for Late Effects. All were at least 18 years old and at least 5 years after treatment. 235 survivors were younger than 18 years old and less than five years after treatment of childhood cancer. We followed them at the University Children Hospital. Of the remaining survivors who had surgery only 95 were followed by surgeons or they refused regullary follow up. There were 72 patients originally from other parts of former Yugoslavia, who were not followed after 1991 when Slovenia became independent state.

The patients were systematically sent to the cardiologist for evaluation, when they came to the outpatient Clinic for late effects. There was no selection.

In 211 survivors complete cardiac evaluation was done. They were treated for childhood cancer at the ages of two month to 18 years (mean 9 years). Regarding the age at diagnosis the patients were divided into three age groups (0–6 years, 7–12 years, 13–18 years). According to the type of malignancy, we divided patients into six groups: patients with leukemia, Hodgkin disease, non-Hodgkin lymphoma, sarcoma, brain tumors and other tumors. The group of other tumors consisted of nephroblastoma, neuroblastoma, hepatoblastoma, retinoblastoma and carcinomas. The treatment modalities were surgery, chemotherapy, radiotherapy or any combination of those. Due to the considerable differences in treatment during different time periods, three groups of patients were formed: from 1968–1978, 1979–1988, 1989–1998. The duration of follow-up ranged from 5 to 32 years (mean 16 years).

See Additional file 1.

### Cardiac evaluation

Cardiac evaluation included history, physical examination, electrocardiography (ECG), exercise testing using a bicycle ergometer, and echocardiography. New York Heart Association (NYHA) functional classification and therapeutic classification applied to dyspnea were used.

A supine standard 12-lead ECG was performed.

A submaximal exercise tolerance test was performed using a bicycle ergometer. Every 3 minutes the workload was increased by 30-, 40-, or 50-W increments as determined by gender and weight. The exercise testing was stopped when the expected heart rate at 75% aerobic efficiency (according to age, sex, and body weight) was reached or due to symptoms suggestive of cardiovascular disorder. During exercise and recovery, ECG, blood pressure and heart rate were measured every 3 minutes. The achieved working capacity was expressed both in absolute terms and as a percentage of predicted values according to standards adjusted for age, sex and body weight. The echocardiographic evaluation consisted of M-mode, two-dimensional, and pulsed, continuous-wave and color Doppler echocardiography. To evaluate the left ventricle, the following parameters were measured: left ventricular end-diastolic and end-systolic dimension and septal and posterior wall thickness at end diastole. The anterior-posterior and superior-inferior diameter of the left atrium were measured from apical 4-chamber view using two-dimensional echocardiography. A color Doppler echocardiography was used to evaluate valvular function. The systolic function of the left ventricle was defined by two parameters: the ejection fraction (EF) and the fractional shortening (FS). The diastolic function of the left ventricle was assessed with pulsed Doppler echocardiography. Peak early mitral blood flow velocity (E) and peak mitral velocity during atrial contraction (A) were measured. The E/A ratio and deceleration time of peak early blood flow velocity (DT) and isovolumic relaxation time (IVRT) were determined. The velocity profile in the right upper pulmonary vein was analysed: an anterograde velocity recorded during ventricular systole (wave S); the anterograde diastolic wave (wave D); and the reverse pulmonary venous flow during atrial contraction (wave A).

### Statistical methods

The following parameters for univariate analysis of various risk factors on cardiac damage were used: age at diagnosis, sex, type of malignancy, time period of treatment, type of treatment, cumulative dose of anthracyclines, concomitant treatment with antracyclines and alkylating agents, dose of chest irradiation and duration of follow up.

The chi-square test was used. P-values of < 0,05 were considered statistically significant.

#### Classification tree analysis

Classification tree analysis is a method of multivariate analysis that allows to study of simultaneous influence of a series of independent variables on one dependent variable [12]. The analysis is performed by successive divisions of the original group of cases into pairs of subgroups, where each division is based on the value of a single independent variable. The variable that produces the purest pair of case subgroups is chosen for a division (division being often referred to as a split). A purity of a case group is measured as a fraction of cases with the same value of the dependent variable: a completely pure group contains cases that have the same outcome. Each of the subgroups in the pair becomes a parent group in the next step of the analysis and is further divided in the same way. The division of cases stops when the group of cases is completely pure or when it contains less than operator-defined minimal number of cases. In our study, the C4.5 [13] program for building classification trees was used. C4.5 allows the setting of several parameters that influence branching and quality of the final classification tree: most notably there is one parameter that determines the smallest number of cases to be included in a single group (mentioned already above in table 1), and another parameter that determines the degree of post-pruning performed. For details please refer to the description in [13]. The optimal values of these parameters were determined using a standard cross-validation method [14-16]. The usual performance measure for classification trees is the accuracy of the tree when predicting the outcome (the value of the dependent variable) on samples not seen during the process of tree building.

Note finally, that since we use an alternative performance criterion, the classification tree obtained the cross-validation procedure outlined above is not expected to provide accurate classification of cases into cardiac damage and non-cardiac damage classes. Instead of using the tree as an accurate predictor, we were interested in analyzing the tree structure and identyfing the risk group where incidence of cardiac damage is significantly higher than the one observed in the population of 211 patients included in the study.

Multivariate analysis with classification tree was not done when specific abnormalities were found in less than ten percent of examinated childhood cancer survivors.

The study was performed in compliance with the Helsinki Declaration with the approval N° 38/11/96 of National Medical Ethics Committee of Slovenia. All the patients gave their approval for the parcipitation in Late Effect Study.

## Results

Among 211 childhood cancer survivors tested for cardiac damage 38 (18%) complained of fatigue, shortness of breath on exercise, palpitations and nonspecific chest pain on exercise (NYHA class 2). Twenty-two patients (4,3%) had high blood pressure.

### Standard Twelve-Lead Electrocardiography

In nineteen (9,2%) survivors the following abnormalities of resting ECG were detected: ST-T wave changes, prolongation of interval QT, right bundle branch block, supraventricular and ventricular premature complexes and supraventricular tachycardia. Electrocardiographic abnormalities were more frequent in patients who were treated with anthracyclines, in particular with high cumulative dose of anthracyclines, although this difference was not statistically significant (p = 0.08)

### Exercise testing

An Exercise testing was performed in 179 patients (85%). In nineteen (10%), the testing was ended due to fatigue and weakness, dyspnea and nonspecific chest pain. In one hundred and two patients (57%) the heart rate increased excessively. The exercise tolerance was below the predicted values in eighty-three patients (46%). In 62% of these patients ECG and echocardiography abnormalities were found.

In only one patient signs of myocardial ishaemia were detected during testing.

The type of malignancy influenced significantly on diminished exercise tolerance in univariate analysis (p = 0.0001). Patients who were treated for brain tumors were at the highest risk for diminished exercise tolerance.

### Echocardiography

#### Size of heart chambers

Thirteen patients (6,9%) had dilated left heart chambers. The cumulative dose of anthracyclines (p = 0.0034) and concomitant treatment with anthracyclines and alkylating agents (p = 0.049) significantly influenced their dimensions.

#### Heart valves and pericardium

In 48 patients (23%) structural or functional damage to the heart valves was found. Most had asymptomatic mitral and tricuspidal valvular thickening with or without mild regurgitation or stenosis. Five patients had asymptomatic moderate regurgitation. There were no patients with severe valvar regurgitation or severe stenosis.

The type of malignancy (p = 0.0006), radiation therapy (p = 0,00001) and concomitant treatment with radiotherapy and anthracyclines (p = 0,037) influenced significantly valvular heart disease in univariate analysis.

In seven patients (3,3%) echocardiographic changes consistent with thickened pericardium were found.

#### Left ventricular systolic function

A left ventricular systolic dysfunction was found in 15 patients (7,1%). The exercise tolerance was below the predicted value in 5 patients (33%) with systolic dysfunction. In one of them, myocardial ischemia was detected during exercise testing.

Nine patients with systolic dysfunction (9/15) had dilated left heart chambers.

Concomitant treatment with anthracyclines and alkylating agents significantly influenced the left ventricular systolic function (p = 0,05).

#### Left ventricular diastolic function

A left ventricular diastolic dysfunction was detected in 59 patients (28,6%). IVRT was measured in 186 patients, 33 of whom had prolonged IVRT. E and A velocities were measured in 199 patients. Thirty-four patients had abnormal ratio E/A. Eight patients had both parameters abnormal. A pattern of abnormal relaxation was found in 25 patients and »pseudonormalisation« in nine patients. The deceleration time of the early wave E was pathological in 72 patients.

Exercise tolerance was below the predicted values in 28 patients (57%) with diastolic dysfunction.

Chemotherapy with anthracyclines (p = 0,013) and therapy with irradiation (p = 0,014) was significant for abnormal ratio E/A but not the prolonged IVRT.

See Additional file 2.

### Multivariate analysis

Decision trees multivariate analysis was performed stepwise analysing independent influence of risk factors on different levels of cardiac damage. First we analysed the impact of above mentioned independent variables on heart valves damage in 208 patients. We used a 10 fold cross validation test mode. The variable that gave the first division was radiotherapy with the heart involved in the field, showing just 14% of patients without radiotherapy against 47% in the radiotherapy group who had heart valve disease. Further division in the radiotherapy group revealed that the patients who were not treated with anthracycline had more valvular damage than those treated with it.

See Figure 1.

**Figure 1:**
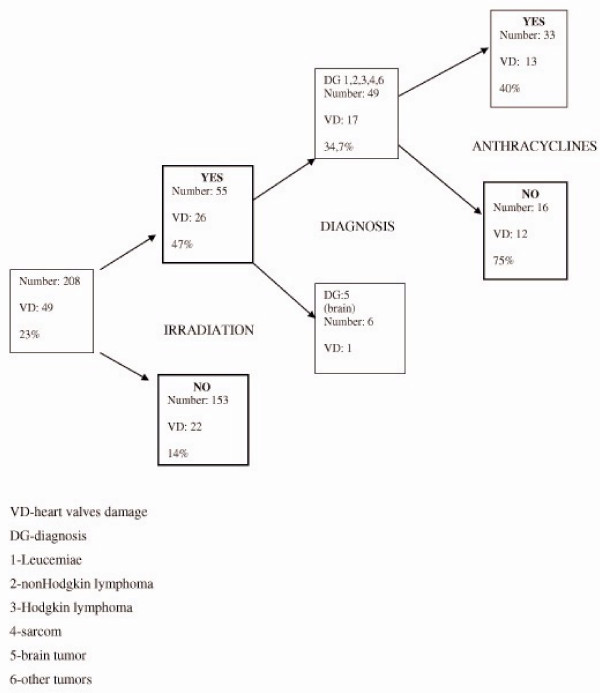
Clasification tree for risk of heart valve disease after treatment of childhood cancer.

The next analyzed outcome was diastolic dysfunction in 59 patients. The variables were the same. The surogate marker for diastolic dysfunction was IVRT. The resulting tree has just one branching and shows that anthracycline treatment was the most important factor for diastolic dysfunction regardless of the cumulative dose.

See Figure 2.

**Figure 2:**
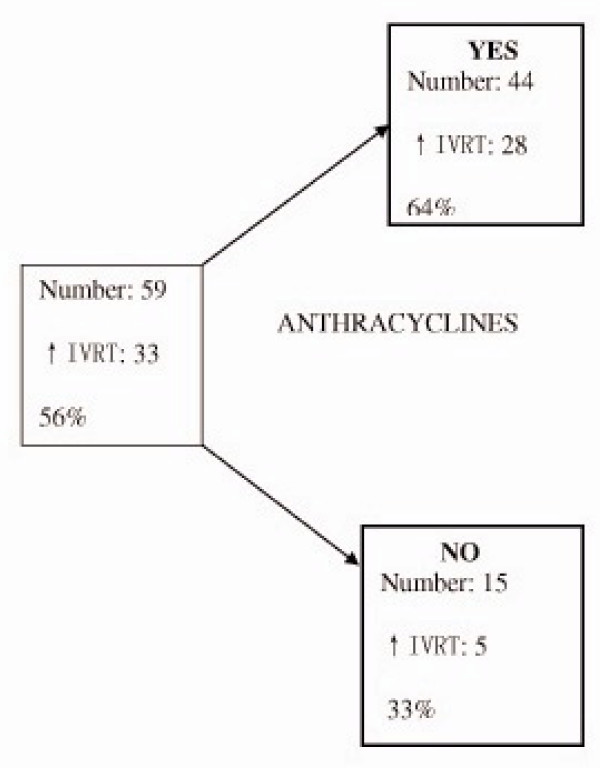
Decision tree for risk of diastolic dysfunction.

The last decision tree analysis was performed using the same variables and the overall cardiotoxicity as the endpoint in 211 patients. Cardiotoxicty as a defined end-point was any abnormality in the structure and/or function of the heart found by our test battery. The resulting tree was highly branched. The period of treatment produced the first division showing that patients treated in the period from 1989 to 1998 were at the highest risk for cardiotoxicity.

See Figure 3.

**Figure 3:**
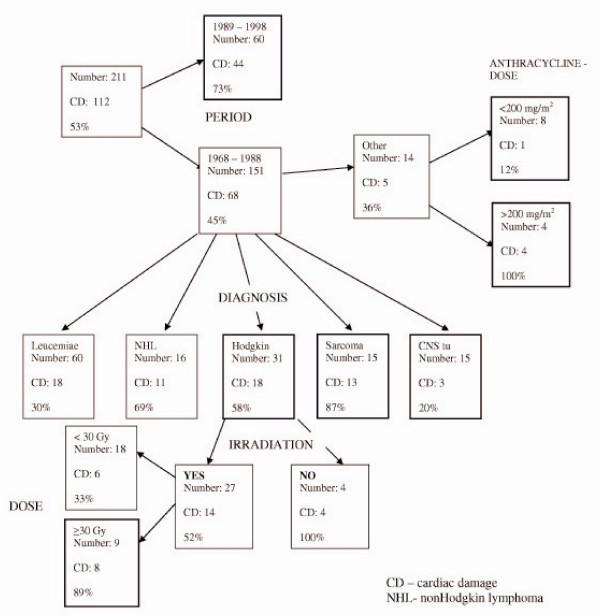
Decision tree for overall cardiac damage after treatment of childhood cancer.

For the patients treated in the period from 1968–88 there was further branching according to type of neoplasm. Patients treated for sarcoma and non-Hodgkin disease were the most affected groups having cardiotoxicity in 87% and 69% respectively. Among patients with Hodgkin disease, those who were treated with irradiation dose above 30 Gy were at highest risk. The group with lowest risk were patients treated for brain tumor (20%).

## Discussion

In recent years, cardiotoxicity after cancer treatment has been a subject of many papers and comprehensive reviews [3,10,17-20]. Most of them studied the effect of anthracycline treatment on cardiac function [4,5,7,21-23]. In many anthracyclines studies, patients treated with irradiation have been excluded. Conversely, in many studies reporting cardiac side effect of irradiation, the patients have not received anthracyclines. In this study of 211 long-term childhood cancer survivors, we have found cardiac damage of various types in more than half of studied subjects. Our study group was heterogenuos according to the type of cancer treated. On the other hand it was a very homogenuos population wise. Moreover, patients were treated and followed-up in a single centre. The statistical method we have used enabeled us to study simultaneous influence of a series of independent variables, such as chemotherapy and radiotherapy, on different aspects of late cardiac sequelae. Our results indicate that different modes of anticancer treatment cause different types of damage of cardiac structure and/or function.

According to our results the time period of the patient's treatment emerged as an important risk factor for injury of the heart. Our finding of multivariate analysis show that patients treated latest, in the years 1989–98 are at highest risk for any injury of the heart. These patients were treated with intensive multiagent chemotherapy. They received larger cumulative doses of anthracycline (more than 200 mg/m^2^) and they received more agressive chemotherapy. They were treated often concomitantly with anthracycline and alkylating agents.

See Additional file 3.

It is reported by some authors in similar studies that female gender is a predictor for cardiac damage. We were not able to confirm this observation with the results of our study.

All our patients with pericardial lesions were asymptomatic. Minor and localised thickening of pericardium has also been observed by others [8,17,24]. The frequency of reported clinically apparent radiation pericarditis following irradiation was 0% to 2,4% [9,24].

The thickening of the heart valves, mostly mitral and aortic, was a frequent finding after radiation therapy, increasing with total doses higher than 30 Gy (23% of all patients). Similar findings were reported by Glanzmann [8,9,24]. In most cases, the valve abnormalities were slight and without haemodynamic consequences. Patients treated with irradiation and without anthracyclines are at the highest risk for heart valve disease which occured in 75% of patients. A possible explanation for this could be that in the time period higher doses of irradiation were used (> 30 Gy), older radiation therapy techniques were available and less effective shielding was used. After a longer observation time (> 20 years) valvular dysfunction have been observed in more patients in our study (30%) than in patients with shorter observation time (< 20 years) although the difference was not statistically significant. In a similar study, valvular heart disease increased from 20% 5 years after irradiation to 60% after 18 years [8]. Patients have been treated with mediastinal radiotherapy and chemotherapy.

Patients in our study treated with a large cumulative dose of anthracycline and those treated concomitantly with anthracyclines and alkylating agents were at the highest risk for systolic dysfunction and enlarged left heart chambers. These results are in concordance with the results reported by other authors [3,5,21,26,27]. However, our cumulative dose at which myocardial toxicity was reported was lower than the doses at which myocardial toxicity was reported by other authors [3,5,10,21]. The group of patients treated with radiotherapy alone seemed to have tolerated the therapy better than the anthracycline group in terms of myocardial function [8,17].

However no statistically significant relationship between the anthracycline dose and abnormal diastolic function was observed. This is in concordance with Sorenson et al [28]. The individual patients may have a lower treshold and developed cardiotoxicity at a dose of anthracycline less than 100 mg/m^2 ^[29,30]. Abnormalities of active myocardial relaxation may occur according to the relative importance of myocyte loss, residual myocyte hypertrophy and interstitial fibrosis in each individual [31,32].

The results of our study showed that exercise tolerance was below the predicted values in almost one half of all survivors (46%). Deterioration of cardiac exercise tolerance is a common finding in children after anticancer chemotherapy, but in most cases it is subclinical [33]. The electrocardiogram and echocardiography revealed abnormalities in more than half of these patients (62%). They could be classified as chronic heart failure class 1 (NYHA).

In multivariance analysis, no specific treatment related factor was associated with lower exercise tolerance. Similar findings were reported by other studies [33]. This would explain the low level of exercise tolerance due to malignant disease and its treatment in childhood. Physical inactivity and obesity in more than one third of our patients may have contribute to worsening of the cardiac function in later years.

We have confirmed the frequent occurence of ECG abnormalities, including changes of ST-T wave, prolongation of QT interval, AV block, right bundle branch block, supraventricular and ventricular premature complexes, supraventricular tachycardia. However, all abnormalities were of little clinical significance. We did not find any significant relation between ECG changes and type of anticancer treatment although 17 out of 19 patients with abnormalities in ECG were treated with anthracyclines. Neither have we found a relation between abnormal systolic or diastolic function and findings on standard ECG.

The results are in concordance with the results reported by other authors [35]. Using a multivariate method of decision trees, we were able to confirm the observation reported by other authors studying cardiac sequelae after treatment of childhood cancer. We have at the same time confirmed the results obtained by decision tree analysis with univariate statistical methods.

## Conclusion

• The frequency of late cardiac effects after childhood cancer treatment in the present study was 53%. One of the patients died of sudden cardiac death 25 years after radiation therapy for Hodgkin's disease. The autopsy revealed myocardial fibrosis.

• Patients treated with large cumulative doses of anthracycline and those treated concomitantly with anthracyclines and alkylating agents are at the highest risk for systolic dysfunction and enlarged left heart chambers.

• Patients treated with anthracyclines are at the highest risk for diastolic dysfunction.

• Patients treated with incidental irradiation of the heart are at the highest risk for heart valves disease.

Our findings demonstrate that the highest risk for any type of cardiac damage after treatment of childhood cancer is present in:

• All patients treated latest, in the years from 1989–98 (73%).

• Among those treated earlier from (1968–78, 1979–88) for Hodgkin's lymphoma treated with irradiation above 30 Gy.

• Patients with sarcoma who were treated in the years from 1968–88.

## Conflict of interest statement

The authors declare that they have no competing interests.

## Authors' contributions

All authors read and approved the final manuscript. VV caried out the patient recruitment, acquisition and interpretation of the data. VV and JJ also drafted the manuscript. UM performed the cardiac evaluation. DD performed the decision tree analysis. BJ is a leader of Late effect study group. She and CK participated in the design of the study, carried out the patient recruitment and gave final approval of the version to be published.

## Pre-publication history

The pre-publication history for this paper can be accessed here:



## Supplementary Material

Additional file 1 Description and values of the independent variables and the dependent variable (last row-cardiac damage) used for univariate and mutivariate analysis. Click here for file

Additional file 2 Factors significantly associated with abnormalities on cardiac evaluation (univariate analysis). Click here for file

Additional file 3 Treatment during three different time intervals. Click here for file
