# Supplementary material for: Cardiac damage after treatment of childhood cancer: A long-term follow-up
Source: BMC Cancer. 2008 May 20;8:141. doi: 10.1186/1471-2407-8-141 (PMC2430718; doi:10.1186/1471-2407-8-141)
Supplement: Additional file 1 — Description and values of the independent variables and the dependent variable (last row-cardiac damage) used for univariate and mutivariate analysis. [file 1471-2407-8-141-S1.doc]

| Variable name | Description |
| --- | --- |
| Sex | Male (113), female (98) |
| Age at diagnosis | 0-6 years (87 pts), 7-12 (57 pts), 13-18 (67 pts) |
| Diagnosis | leukemia (77 pts), non-Hodgkin lymphoma (22 pts), Mb. Hodgkin (54 pts), Sarcomas (25 pts), CNS tumors (17 pts), others (16 pts) |
| Time period of treatment | 1968-1978 (39 pts), 1979-1988 (112 pts), 1989-1998 (60pts) |
| Surgery | Yes (115), no (96) |
| Radiotherapy | Yes (166), no (45) |
| Cumulative radiation dose | <30 Gy (30 pts), >30 Gy (26 pts) |
| Chemotherapy | Yes (191), no (12) |
| Anthracycline | Yes (146), no (65) |
| Cumulative dose of anthracycline | <200 mg/m^2^ (62 pts) 200-400 mg/m^2^ (70 pts), >400 mg/m^2^ ( 14 pts) |
| Cardiac damage | Yes (112), no (99) |
