# Supplementary material for: Cardiac damage after treatment of childhood cancer: A long-term follow-up
Source: BMC Cancer. 2008 May 20;8:141. doi: 10.1186/1471-2407-8-141 (PMC2430718; doi:10.1186/1471-2407-8-141)
Supplement: Additional file 2 — Factors significantly associated with abnormalities on cardiac evaluation (univariate analysis). [file 1471-2407-8-141-S2.doc]

|  | Risk factor | p-value |
| --- | --- | --- |
| ECG | Anthracyclines | 0,08 |
| Achieved exercise tolerance | Type of malignancy | 0,0001 |
| Size of heart chambers | Cumulative dose of anthracyclines | 0,0034 |
|  | Anthracyclines and alkylating agents | 0,049 |
| Heart valves | Type of malignancy | 0,0006 |
|  | Irradiation of the heart | 0,00001 |
|  | Irradiation and anthracyclines | 0,037 |
| Systolic function | Anthracyclines and alkylating agents | 0,05 |
| E/A | Irradiation | 0,014 |
|  | Anthracyclines | 0,012 |
| Cardiac damage | Age at diagnosis | 0,034 |
|  | Type of malignancy | 0,005 |
|  | Time period of treatment | 0,038 |
|  | Irradiation of the heart | 0,041 |
|  | Anthracyclines | 0,03 |
|  | Cumulative dose of anthracyclines | 0,0067 |
|  | Anthracyclines and alkylating agents | 0,00136 |
