# Supplementary material for: Cardiac damage after treatment of childhood cancer: A long-term follow-up
Source: BMC Cancer. 2008 May 20;8:141. doi: 10.1186/1471-2407-8-141 (PMC2430718; doi:10.1186/1471-2407-8-141)
Supplement: Additional file 3 — Treatment during three different time intervals. [file 1471-2407-8-141-S3.doc]

| **Treatment modality** | **1968 to 1978**  **number of pts (%)** |  | **1979 to 1988**  **number of pts (%)** | **1989 to 1998**  **number of prs (%)** |
| --- | --- | --- | --- | --- |
| **Chemotherapy with anthracyclines** | 13/39 (33) |  | 83/112 (74) | 50/60 (83) |
| **Anthracyclines and alkylating agents** | 11/39 (28) |  | 37/75 (49) | 50/60 (83) |
| **Heart irradiation** | 7/39 (18) |  | 26/112 (23) | 23/60 (38) |
| **Dose of irradiation** | 1/7 (14) | **< 30 Gy** | 17/26 (65) | 11/23 (48) |
|  | 6/7 (86) | **≥ 30 Gy** | 9/26 (35) | 12/23 (52) |
| **Anthracyklines and heart irradiation** | 0/39 |  | 15/112 (13) | 18/60 (30) |
| **Cumulative**  **dose of** | 6/13 (46) | **< 200mg/m^2^** | 40/83 (48) | 16/50 (32) |
| **anthracyclines** | 5/13 (38) | **200-400mg/m^2^** | 34/83 (41) | 30/50 (60) |
|  | 2/13 (15) | **> 400 mg/m^2^** | 9/83 (11) | 4/50 (8) |
